# Supplementary material for: A study protocol for the validation of a prognostic model with an emphasis on modifiable factors to predict chronic pain after a new episode of acute- or subacute nonspecific idiopathic, non-traumatic neck pain presenting in primary care
Source: PLoS One. 2023 Jan 17;18(1):e0280278. doi: 10.1371/journal.pone.0280278 (PMC9844852; doi:10.1371/journal.pone.0280278)
Supplement: Attachment — Submitted filename: PONE-D-22-19562.docx [file pone.0280278.s001.docx]

**PLOS ONE	PONE-D-22-19562**

A study protocol for the validation of a prognostic model with an emphasis on modifiable factors to predict chronic pain after a new episode of acute- or subacute nonspecific idiopathic, non-traumatic neck pain presenting in primary care.

**I highly commend the authors for their high quality work and the potential prospect impact on health care and clinical decision making in the care of neck pain and prediction of potential factors that may lead to chronicity, in deed this is a great valuable research efforts.**

***However, the following is my humble criticism***

**Objectives**

**You wrote ;**

“*The primary objective of this study is to identify which modifiable and non-modifiable factors are independent predictors of the development of chronic pain in patients with acute- or subacute nonspecific idiopathic, non-traumatic neck pain, and secondly, to combine these to develop and internally validate a prognostic prediction model”.*

*With regards to the objectives I fully I agree with the authors . Their objectives are legitimate and rational , and these kind of studies is needed to improve the clinical work of physiotherapist.

***However,*** I should point out that because there is a synchronized positive relationship between pain, and fear related illness behaviors in acute sage most of the psychological factors cannot be modifiable at acute stage because it is natural response to pain. However, this relationship may vanish or persist in most people following subacute stage and may be reinforced in others due to selected environmental biopsychosocial factors as you indicated. The role of the psychological factors mainly fear of/ anticipation of pain and catastrophizing continue to develop and reinforce the pain and related behavior beyond the subacute stage, so the argument that may arise her is whether the emphasis should be directed to screen for the most frequent reinforcers found in the chronic stage and then to be used later in acute or subacute stage as prognosis predictors so that the therapist screen to identify and modify.

**Study Settings**

You have large ***Number*** of physiotherapist included in the study this may have advantage or disadvantage specifically, related to these individual knowledge and understanding of the biopsychological factors, and skills in screening, or treatment implementation of these knowledge in their workplace this may need to elaborated on don you agree .

**Participant**

“The patients are at least 18 years or older”

Neck pain is common among adults, although it can occur at any age. Are you a wear of the Global Burden of Diseases 2017 study, it demonstrated that the point prevalence of neck pain peaked during the middle ages with the highest burdens in people age 45–49 and 50–54 for men and women, respectively.

*Kazeminasab, S., Nejadghaderi, S.A., Amiri, P. et al. Neck pain: global epidemiology, trends and risk factors. BMC Musculoskelet Disord 23, 26 (2022). https://doi.org/10.1186/s12891-021-04957-4*

***Therefore,*** including young participant starting from age 18 years may not reflect the day today patient volume in any physical therapy clinical setting. In addition, young individuals are motivated, highly active and may have different biopsychosocial factors than the adults or senior individual.

You may need to reflect on the young age pain behavior psychology if you have a huge number of young individuals or eliminate young individuals as a sub population in your study.

***Item 4*** “If the patient has had neck pain before, the patients must be relatively free from symptoms for at least three months”

**My question** **to you** is how would you assume that this patient has no learning effect from his previous episodes pain related fear experience and behavior , I though you needed an individuals with totally knew pain experience to be able to demonstrate a good predicting tool.

I have reviewed the Delphi survey and your systematic survey **I think I perceived that unhealthy life style and physical inactivity were considered separate variables,**

**I also did not find any mentioned to somatization in your factors**, this was mentioned earlier by the Delphi survey could you define it and further elaborate why it was not included

- ***Her is another confusing problem for the prognostic tool you are about to produce and please allow me to elaborate and explain my worries and its all centered around Kinesiophobia.***

Kinesiophobia defined as “an excessive, irrational, and debilitating fear of physical movement and activity resulting from a feeling of vulnerability due to painful injury or reinjury”

It is by itself a central factor in the process of pain developing from acute to chronic stages, The Cognitive Fear Avoidance Model describes that very clearly , it also linked it strongly to catastrophizing, re/injury, avoidance behaviour, and on the long run inactivity, disability, depression, low self-esteem and increasing the risk for a wide range of health problems, functional decline and premature death.

***My question*** ***is***

You have included so many independent factors that have high potential prognostic ability alone, so it become very difficult and confusing to understand the weighting of theses variables to the final prediction scores on your prognostic tool while in reality each one of which is so representative and highly weighted predictor itself such as kinesiophobia , depression, anxiety, low self-esteem. Need to elaborate a bit more on this.

**Discussion**

Line 338-350	I found the following statement very confusing and not helpful to justify the objectives of the study

**338**. The given therapy may influence the outcome and the accuracy and transportability of the model to be developed.

**340. T**he patients receive standard care based on the Dutch Physiotherapy Guideline for neck pain.

**344-345.** We will have no information on this form of treatment during this study; however, it could influence the outcome.

**You wrote** “Nevertheless, we consider the impact on our study findings to be minimal, given ……..”

In their conclusion *Carroll LJ, Hogg-Johnson S, van der Velde G, Haldeman S, Holm LW, Carragee EJ, Hurwitz EL, Côté P, Nordin M, Peloso PM, Guzman J, Cassidy JD. Course and Prognostic Factors for Neck Pain in the General Population: Results of the Bone and Joint Decade 2000 –2010 Task Force on Neck Pain and Its Associated Disorders. Eur Spine J. 2008 Apr;17(Suppl 1):75–82. doi: 10.1007/s00586-008-0627-8. Epub 2008 Feb 29. PMCID: PMC2271093.* “General exercise was not prognostic of better outcome; however, several psychosocial factors were prognostic of outcome”.

In their discussion they also have specifically stated that **“ *Prognosis may also depend on whether or not the exercises themselves were designed to impact the***

***neck and shoulder areas”.***

**My question**

since the author of the above study concluded *only on general exercises*, but have raised the *potential value of specific designed exercise* then this raise the concerns about the type of approach used in these multicenter and by the physiotherapist skills and preferences involved in this study .

The benefit and impact of specific approaches as the directed self treatment exercise for the neck used by well trained therapist such as Maitland, McKenzie, or Shacklock M neurodynamic and others on the outcomes has ben well documented such techniques has prognostic value against psychological factors and chronicity.

For example directional preference exercise by the McKenzie approach help the patient face his one fear of painful movement direction while rehearsing in this direction which help centralized improve and prevent the next episodes of pain and fast return to work.

**Line 348-350**

**You wrote**

“Thereby, we will report the physiotherapy treatment

the patient received and discuss the possible impact on our study findings (TRIPOD 5C) but do not include the different treatments as a predictor in our model” .

I think you may need to reconsider these statement in light of the variation of approaches among theses centers to reveal the impact of these specific neck exercise approach on modifying the psychological factors
